# Supplementary material for: The relationship between disease activity and quality of life in rheumatoid arthritis patients: a network analysis
Source: PeerJ. 2025 Aug 21;13:e19907. doi: 10.7717/peerj.19907 (PMC12375295; doi:10.7717/peerj.19907)
Supplement: Supplemental Information 1 [file peerj-13-19907-s001.docx]

**Table S1.** The weighted adjacency matrix for networks without (left) and with (right) depressive symptoms

|  | PF | RP | BP | GH | VT | SF | RE | MH |
| --- | --- | --- | --- | --- | --- | --- | --- | --- |
| PF |  | 0.463 | -0.081 | 0.168 | 0.031 | 0.194 | 0.072 | 0 |
| RP | 0.547 |  | 0 | 0.096 | 0 | -0.162 | 0 | 0 |
| BP | -0.404 | 0.235 |  | 0 | 0.070 | 0.022 | 0 | 0 |
| GH | 0.077 | 0.327 | 0.036 |  | 0.157 | 0.280 | 0.135 | 0 |
| VT | 0.046 | -0.058 | 0.082 | 0.099 |  | 0 | 0 | 0.640 |
| SF | 0.073 | 0 | 0.098 | 0.236 | 0.046 |  | 0.066 | 0.465 |
| RE | 0.247 | -0.075 | 0.155 | 0.348 | 0 | 0 |  | 0 |
| MH | -0.092 | 0 | -0.166 | 0 | 0.702 | 0.325 | 0.062 |  |

Note: PF: physical function; RP: role-physical; BP: bodily pain; GH: general health; VT: vitality; SF: social function; RE: role-emotional; MH: mental health.
