# Supplementary material for: The relationship between disease activity and quality of life in rheumatoid arthritis patients: a network analysis
Source: PeerJ. 2025 Aug 21;13:e19907. doi: 10.7717/peerj.19907 (PMC12375295; doi:10.7717/peerj.19907)
Supplement: Supplemental Information 2 — Gray boxes indicate EI that does not differ significantly, while black boxes indicate EI that differs significantly. The number in the white boxes (i.e., diagonal line) represents the value of EI. [file peerj-13-19907-s002.pdf]

a

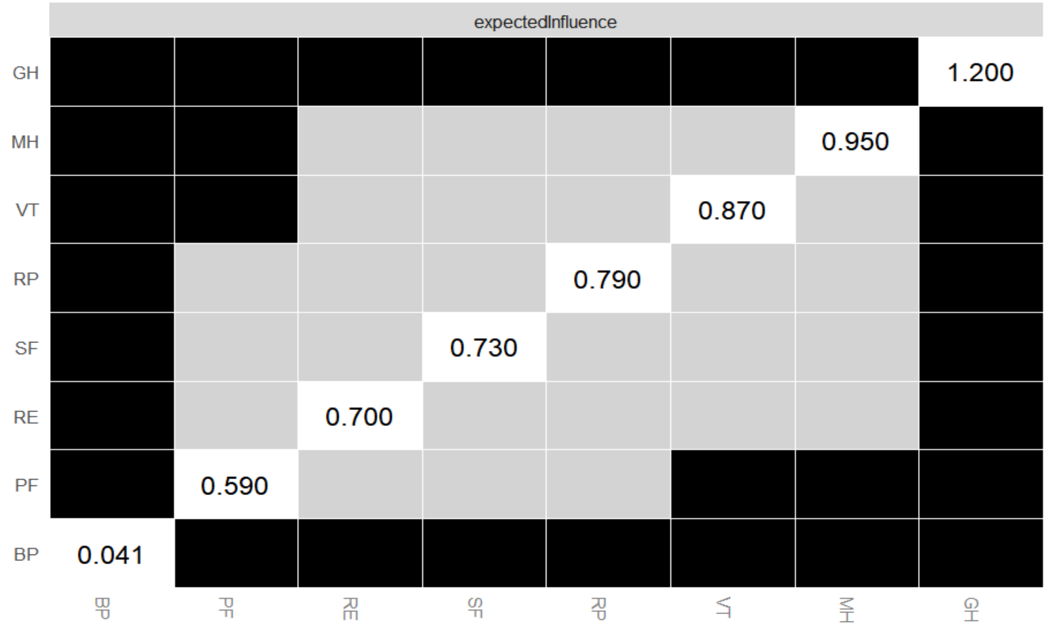

expectedInfluence
GH
MH
VT
RP
SF
RE
PF
BP
1.200
0.950
0.870
0.790
0.730
0.700
0.590
0.041
BP
PF
RE
SF
RP
VT
MH
GH

b

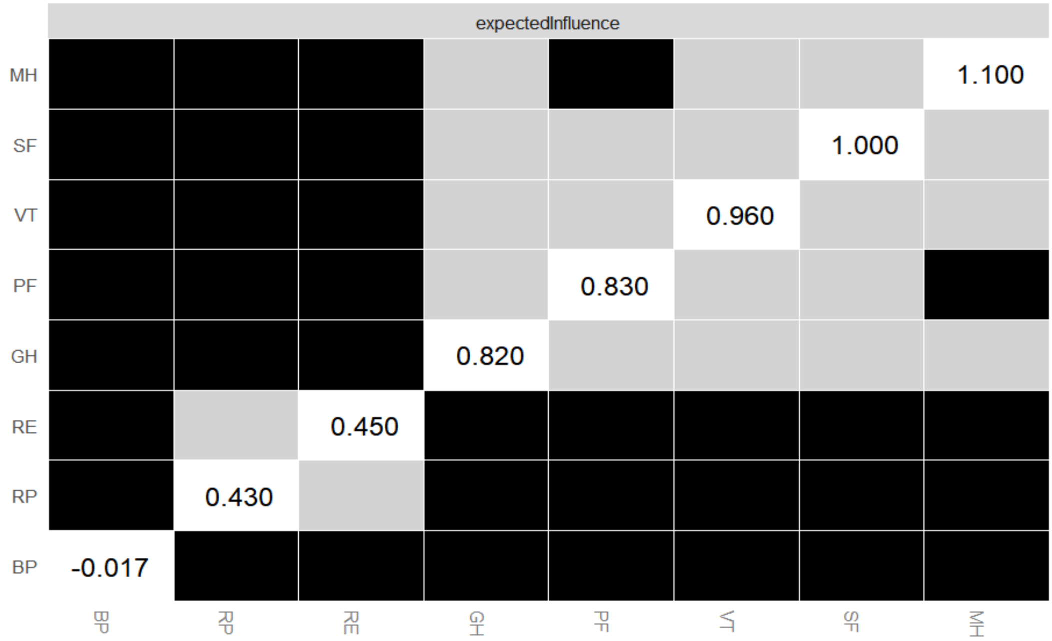

expectedInfluence
MH
SF
VT
PF
GH
RE
RP
BP
1.100
1.000
0.960
0.830
0.820
0.450
0.430
-0.017
BP
RP
RE
GH
PF
VT
SF
MH
