# Supplementary figures and images for: The relationship between disease activity and quality of life in rheumatoid arthritis patients: a network analysis
Source: PeerJ. 2025 Aug 21;13:e19907. doi: 10.7717/peerj.19907 (PMC12375295; doi:10.7717/peerj.19907)

a

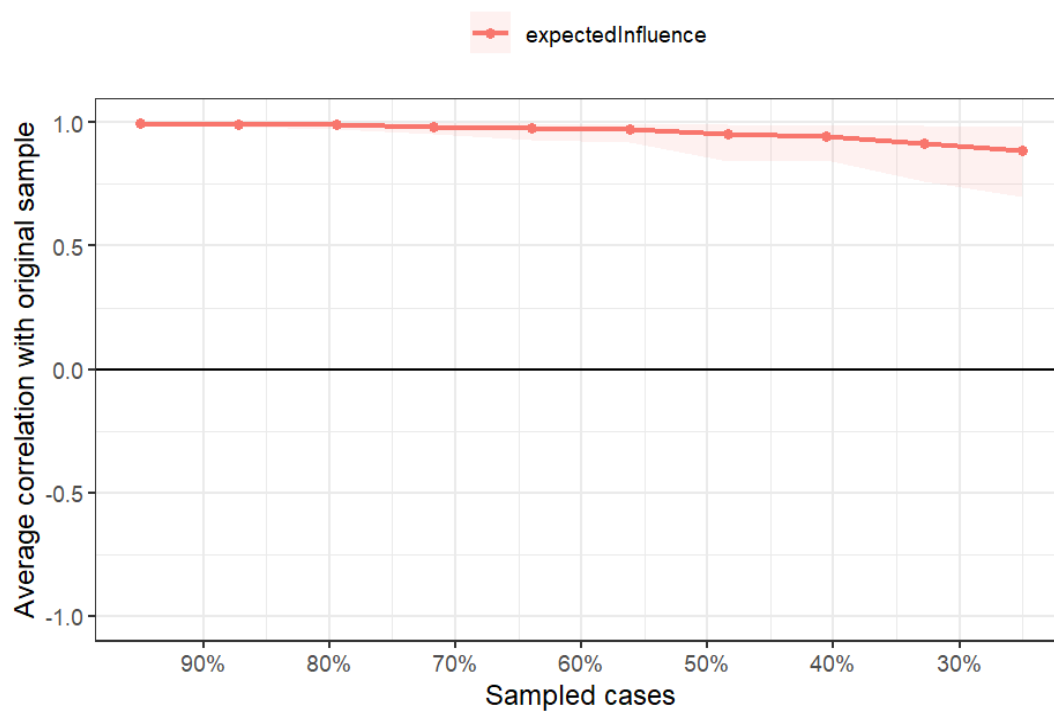

b

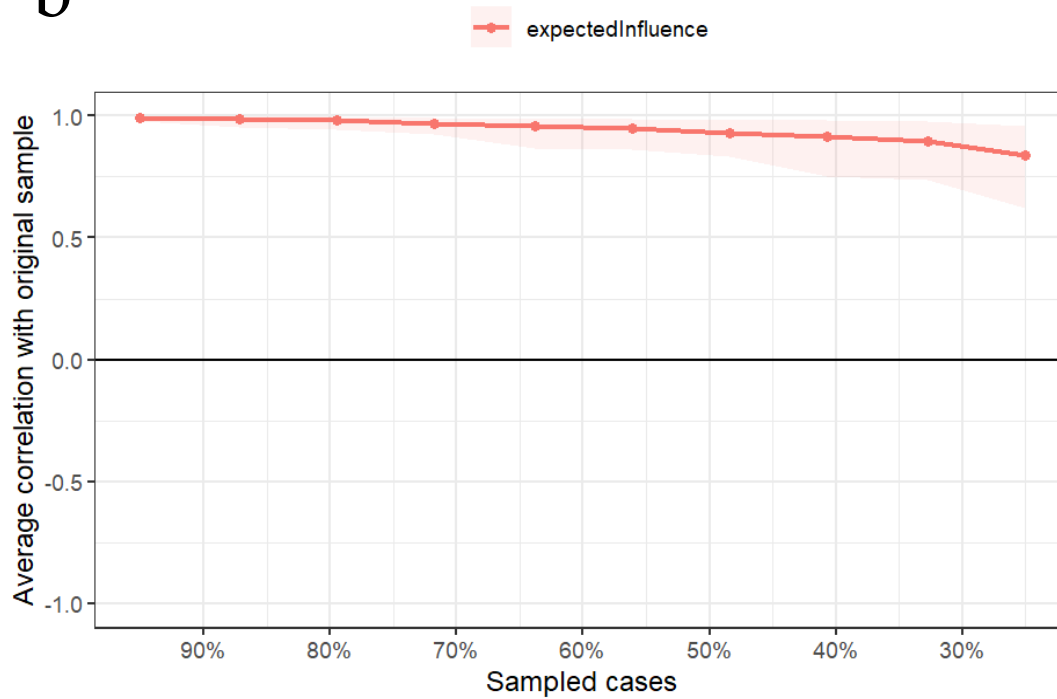

Supplement: Supplemental Information 3 — The x-axis represents the percentage of cases from the original sample used in each step. The y-axis represents the average correlations between the original network’s centrality indices and the re-estimated networks’ centrality indices after excluding increasing percentages of cases. [file peerj-13-19907-s003.pdf]

a

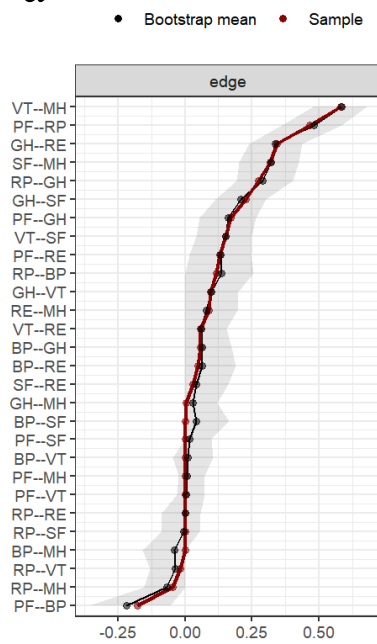

b

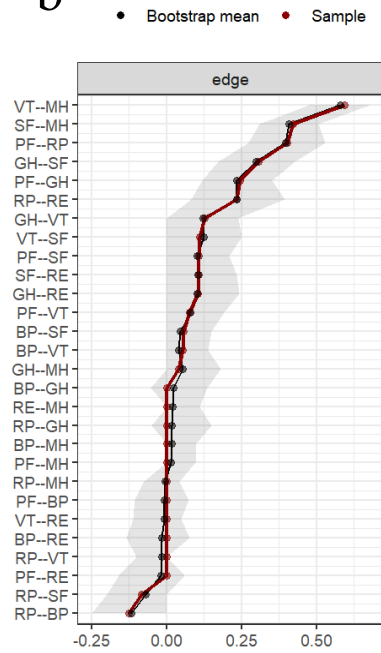

Supplement: Supplemental Information 4 — The red line represents the margin as estimated in the sample. The grey indicates the 95% bootstrapped confidence interval. The x-axis represents the edges, while the grey lines indicate specific edges along the y-axis. [file peerj-13-19907-s004.pdf]
