# Supplementary material for: The relationship between disease activity and quality of life in rheumatoid arthritis patients: a network analysis
Source: PeerJ. 2025 Aug 21;13:e19907. doi: 10.7717/peerj.19907 (PMC12375295; doi:10.7717/peerj.19907)
Supplement: Supplemental Information 6 [file peerj-13-19907-s006.docx]

STROBE Statement

| **Title and abstract** | 1 | The relationship between disease activity and quality of life in rheumatoid arthritis patients: a network analysis |
| --- | --- | --- |
| **Introduction** |  |  |
| Backgrounds | 2 | The relationship between disease activity and quality of life (QoL) in RA patients was explored using network analysis. The focus of network analysis has recently shifted from studying individual groups to comparing the network structures of different subgroups. RA patients with depressive symptoms generally have lower QoL scores, so we compared the QoL networks of RA patients with and without depressive symptoms to test for differences |
| Objectives | 3 | This study aims to (1) compare whether there is a difference in the structure of the QoL network between RA patients with and without depressive symptoms and (2) explore the relationship between disease activity and QoL in RA patients |
| **Methods** |  |  |
| Study design | 4 | Cross-sectional Studies |
| Setting | 5 | 424 patients with RA were recruited from the First Affiliated Hospital of Anhui Medical University. The survey period spanned from January 2024 to July 2024 |
| Participants | 6 | The inclusion criteria were as follows: (1) a diagnosis based on the 2010 American College of Rheumatology (ACR)/European League Against Rheumatism (EULAR) classification criteria and scoring system for RA; (2) informed consent and voluntary participation in the study; (3) age of 18 years or older; and (4) the ability to communicate in Mandarin and complete the questionnaire independently or with assistance from the investigator |
| Measurements | 7 | Including general information, Hospital Anxiety and Depression Scale (HADS), 36-item Short-Form Health Survey (SF-36), and Disease Activity Score 28 joints (DAS8) |
| Study size | 8 | Including 424 patients |
| Statistical methods | 9 | 1. Descriptive analyses of the variables were performed using SPSS 26.0. Network analyses were performed using R software (4.0.0) |
|  |  | 1. The "qgraph" package is used to visualize the network |
|  |  | 1. The "NetworkComparisonTest" package was used to detect if there was a difference between the two networks |
|  |  | 1. Using the "flow" function in the "qgraph" package to explore the relationship between disease activity and QoL |
| **Results** |  |  |
| Main results | 10 | 1. Differences in QoL network structure between RA patients with and without depressive symptoms |
|  |  | 1. The domain of PF (physical function) was most strongly associated with disease activity in RA patients |
| **Discussion** |  |  |
| Key results | 11 | Disease activity is strongly associated with QoL in RA patients, especially in the domain of PF, and the structure of the QoL network changes in the presence of depressive symptoms in RA patients |
| **Other information** |  |  |
| Funding | 12 | This work was supported by the Key Scientific Research Foundation of the Education Department of Anhui Province (No. 2023AH050601) |
